# Pharmacogenetic Study of Deferasirox, an Iron Chelating Agent

**DOI:** 10.1371/journal.pone.0064114

**Published:** 2013-05-30

**Authors:** Ji Won Lee, Hyoung Jin Kang, Ji-Yeob Choi, Nam Hee Kim, Mi Kyung Jang, Chang-Woo Yeo, Sang Seop Lee, Hyery Kim, June Dong Park, Kyung Duk Park, Hee Young Shin, Jae-Gook Shin, Hyo Seop Ahn

**Affiliations:** 1 Department of Pediatrics, Seoul National University College of Medicine, Seoul, Republic of Korea; 2 Cancer Research Institute, Seoul National University College of Medicine, Seoul, Republic of Korea; 3 Department of Biomedical Sciences, Graduate School of Seoul National University, Seoul, Republic of Korea; 4 Department of Pharmacology and Pharmacogenomics Research Center, Inje University College of Medicine, Busan, Republic of Korea; Institut national de la santé et de la recherche médicale (INSERM), France

## Abstract

Transfusion-associated iron overload induces systemic toxicity. Deferasirox, a convenient long acting oral agent, has recently been introduced in clinical practice with a promising efficacy. But there are some patients who experience drug-related toxicities and cannot tolerate it. To investigate effect of genetic variations on the toxicities and find optimal target population, we analyzed the genetic polymorphisms of UDP-glucuronosyltransferase 1A (UGT1A) subfamily, multi-drug resistance-associated protein 2 (MRP2) and breast cancer resistance protein (BCRP). A total of 20 functional genetic polymorphisms were analyzed in 98 patients who received deferasirox to reduce transfusion-induced iron overload. We retrospectively reviewed the medical records to find out the drug-related toxicities. Fifteen (15.3%) patients developed hepatotoxicity. Patients without wild-type allele carrying two MRP2 haplotypes containing −1774 del and/or −24T were at increased risk of developing hepatotoxicity compared to patients with the wild-type allele on multivariate analysis (OR = 7.17, 95% CI = 1.79–28.67, *P* = 0.005). Creatinine elevation was observed in 9 patients (9.2%). Body weight ≥40 kg and homozygosity for UGT1A1*6 were risk factors of creatinine elevation (OR = 8.48, 95% CI = 1.7–43.57, *P* = 0.010 and OR = 14.17, 95% CI = 1.34–150.35, *P* = 0.028). Our results indicate that functional genetic variants of enzymes to metabolize and transport deferasirox are associated with drug-related toxicities. Further studies are warranted to confirm the results as the pharmacogenetic biomarkers of deferasirox.

## Introduction

Red blood cell (RBC) transfusion in pediatric patients with cancer and hematologic disease is necessary to maintain body RBC pool. Because human has no biological mechanisms to remove the excess iron, patients who are transfusion-dependent are at risk of iron overload. Free iron catalyzes the conversion of reactive oxygen species intermediates to highly toxic free radicals which may mediate tissue injury [1]. It is known that transfusion-associated iron overload is associated with the increase of the treatment-related toxicity. This is particularly cases with an intensive therapy such as hematopoietic stem cell transplantation (HSCT) [2], [3], [4], [5], [6], [7].

It is highly probable that iron chelation may reduce the iron-induced tissue toxicity and thereby improve the short- and long-term treatment outcome. Our previous report showed that an elevated serum ferritin level was associated with both an increased treatment-related mortality and a decreased survival. In addition, it also suggested that the iron chelating therapy had a benefit of improving the outcomes of HSCT [5].

Deferoxamine has been the standard drug for iron chelation therapy over the past four decades. However, its major disadvantage is non-compliance of patients, because it needs an 8- to 12-hr parenteral administration since it has a short half-life and a very poor oral bioavailability [8], [9], [10], [11]. A more convenient oral iron chelator, deferasirox, has recently become available showing promising efficacy [12], [13], [14], [15], [16], [17]. Many studies have shown that deferasirox has an acceptable profile of safety and tolerability. Nevertheless, it has also been reported that some patients experienced drug-related toxicities and could not tolerate it. The most common side effects of deferasirox were gastrointestinal disturbance and skin rash, but they also include agranulocytosis, creatinine elevation and hepatitis [13], [15], [18], [19], [20], [21].

Pharmacogenetics has been used widely to predict individual responses or toxicities to drugs by investigating genetic differences in metabolic pathways [22]. However, there has been no data published so far showing the relationship between the genetic polymorphisms and the side effects of deferasirox. As it is known that deferasirox is mainly metabolized by glucuronidation and eliminated into the bile through multidrug resistance protein 2 (MRP2) [23], the effect and toxicity of deferasirox may be influenced by the variability of UDP-glucuronosyltransferase, MRP2 and breast cancer resistance protein (BCRP) which are important for intestinal drug absorption and hepatic drug elimination [24]. In this study, we analyzed the genetic polymorphisms of UDP-glucuronosyltransferase 1A (UGT1A) subfamily, MRP2 and BCRP to predict toxicities and find optimal target population for deferasirox treatment in pediatric patients.

## Materials and Methods

### Patients

Patients who received deferasirox because of transfusion-associated iron overload were enrolled for this study. Transfusion-associated iron overload was defined as ferritin ≥1,000 ng/mL in patients who needed RBC transfusions over 8 units per year.

### Ethics

This study was approved by the Institutional Review Board of Seoul National University Hospital (H-0709-011-217) and registered at www.clinicaltrials.gov (NCT01623895). Written informed consents were obtained for all patients from their parents on the behalf of the children participants.

### Definition of drug-related toxicities

During the administration of deferasirox, we examined complete blood count and serum chemistry. We retrospectively reviewed the medical records to find out the drug-related toxicities. Drug-related hepatotoxicity was defined as increased AST or ALT >5×ULN or increased bilirubin >3×ULN which was thought to be caused by deferasirox, and creatinine elevation was defined as serum creatinine level increase more than 50% above baseline.

### Genotyping

Genomic DNA was extracted from peripheral blood cells using QIAamp DNA Blood Mini Kit (Qiagen, Chatsworth, California, USA). Functional genetic variations of BCRP (34G>A, 8191C>T, 8825C>A), MRP2 (−1774delG, −24C>T), UGT1A1 (−3279T>G, −39insTA, 211G>A, 686C>A), UGT1A3 (17A>G, 31T>C, 81G>A, 133C>T, 140T>C, 477A>G), UGT1A7 (387T>G, 391C>A, 392G>A, 622T>C), and UGT1A9 (−118insT) were determined using either direct sequencing or pyrosequencing methods as previously described [25], [26], [27].

Individual haplotypes were estimated from genotype data by the Bayesian method using the PHASE program (version 2.0.2) [28]. Pairwise linkage disequilibrium (LD) between the two alleles was estimated as relative disequilibrium (D’) from estimated haplotype data [29].

### Statistical methods

The Hardy-Weinberg equilibrium was assessed by the chi-square test with df = 1 for all tested single nucleotide polymorphisms (SNPs). Clinical data (body weight, gender, ferritin level and dose of deferasirox), genotype and haplotype were used as independent variables to predict the occurrence of toxicities. We performed a univariate analysis with each genotype and haplotype. Then multivariate analysis was done with genotype/haplotype of which *P-*value was less than 0.2 on univarate analysis. The above-stated clinical variables were used as confounding factors for multivariate analysis. A logistic regression model was used to estimate the odds ratios (OR) and 95% confidence interval for the risk of toxicities. SPSS version 19.0 was used for all statistical analyses, and statistical significance was accepted when *P*-value was less than 0.05.

## Results

### Clinical characteristics of patients

A total of 98 patients (63 male and 35 female) were enrolled. The median age was 9.0 (1.0–23.0) years and the median body weight was 27.4 (8.8–80.0) kg. The diagnoses were acute leukemia in 20 (20.4%), lymphoma in 4 (4.1%), solid tumor in 63 (64.3%), aplastic anemia in 5 (5.1%), and other hematologic disease in 6 (6.1%) patients. Patients received median 29.4 (17.9–34.1) mg/kg of deferasirox as an initial dose, and the median ferritin level at the initiation of deferasirox was 1,943 (1,025–11,032) ng/mL. Sixty-one (62.2%) patients were on their chemotherapy schedule during the use of deferasirox.

### Pharmacogenetic analysis

Genotypes of the 20 candidate loci and haplotypes in 98 patients are summarized in Table S1. All of the genotypes were in Hardy–Weinberg equilibrium (*P*>0.05).

### Hepatotoxicity

Fifteen patients (15.3%) developed hepatotoxicity at a median 60 (13–198) days after the administration. The hepatotoxicity was increased AST/ALT >5×ULN in 14 patients, and increased bilirubin >3×ULN in 1 patient.

Risk factor analyses were performed and the results are summarized in Table I. Risk of hepatotoxicity was significantly increased as non wild-type allele of MRP2 haplotype increased (*P* for trend = 0.013). On univariate analysis, patients without wild-type allele carrying two MRP2 haplotypes containing −1774 del and/or −24T were at increased risk of hepatotoxicity compared to patients with the wild-type allele (OR = 4.92, 95% CI = 1.52–15.90, *P* = 0.008). The effect of MRP2 haplotype maintained the statistical significance on multivariate analysis (OR = 7.17, 95% CI = 1.79–28.67, *P* = 0.005).

**Table 1:** Risk factor analysis of hepatotoxicity.

Clinical or genetic factor	Hepatotoxicity^a^		Univariate analysis		Multivariate analysis^b^	
	Yes	No	OR	*p*	OR	*p*
**Median body weight, kg (range)**	16.2 (8.8–70.0)	30.7 (10.0–80.0)	0.97 (0.93–1.01)	0.128	0.97 (0.94–1.01)	0.145
**Gender, No. (%)**				0.707		0.756
Male	9 (60.0)	54 (65.1)	1.00 (reference)		1.00 (reference)	
Female	6 (40.0)	29 (34.9)	1.24 (0.40–3.83)		1.21 (0.37–4.00)	
**Median ferritin level, ng/mL (range)**	1,720 (1,160–3,128)	1,947 (1,025–11,032)	1.00 (1.00–1.00)	0.137	1.00 (1.00–1.00)	0.134
**Median dose of deferasirox, mg/kg (range)**	27.8 (17.9–34.1)	29.8 (17.9–34.1)	0.90 (0.76–1.04)	0.149	0.88 (0.76–1.02)	0.097
**MRP2 haplotype, No. (%)**				**0.008**		**0.005**
Haplotypes including wild–type	5 (33.3)	59 (71.0)	1.00 (reference)		1.00 (reference)	
No wild type	10 (66.7)	24 (28.9)	4.92 (1.52–15.90)		7.17 (1.79–28.67)	

a Hepatotoxicity was defined as increased AST or ALT >5×ULN or increased bilirubin >3×ULN.

b Adjusting for body weight, gender, ferritin level, and dose of deferasirox.

All of the 15 patients showed decreased AST/ALT and bilirubin (AST/ALT ≤5×ULN and bilirubin ≤1×UNL) at median 9.5 (2–19) days after discontinuation of deferasirox.

### Creatinine elevation

There were 9 (9.2%) patients whose serum creatinine level increased more than 50% above baseline at a median time of 49 (14–128) days after the start of deferasirox. The median age of patients with creatinine elevation was 14.1 (2.8–16.3) years.

Risk factor analyses were performed for creatinine elevation and the results are represented in Table II. The risk of creatinine elevation was higher in patients with a greater body weight when the body weight was analyzed as a continuous variable (OR = 1.05, 95% CI = 1.01–1.10, *P* = 0.010 in univariate analysis and OR = 1.05, 95% CI = 1.01–1.10, *P* = 0.016 in multivariate analysis). When the body weight was divided into categorical variable based on a cut-off value of 40 kg, creatinine elevation occurred more commonly in patients with a body weight of ≥40 kg (22.6%) compared to those with a body weight of <40 kg (3.0%) (OR = 8.48, 95% CI = 1.7–43.57, *P* = 0.010). Patients with homozygote of UGT1A1*6 had 14.17-times higher risk of creatinine elevation than the other patients on multivariate analysis (OR = 14.17, 95% CI = 1.34–150.35, *P* = 0.028).

**Table 2:** Risk factor analysis of creatinine elevation.

Clinical or genetic factor	Creatinine elevation^a^		Univariate analysis		Multivariate analysis^b^	
	Yes	No	OR	*p*	OR	*p*
**Median body weight, kg (range)** c	51.3 (11.0–77.0)	25.8 (8.8–80.0)	1.05 (1.01–1.10)	**0.010**	1.05 (1.01–1.10)	**0.016**
**Gender, No. (%)**				0.876		0.835
Male	6 (66.7)	57 (64.0)	1.00 (reference)		1.00 (reference)	
Female	3 (33.3)	32 (36.0)	0.89 (0.21–3.80)		1.19 (0.23–6.11)	
**Median ferritin level, ng/mL (range)**	1,926 (1,236–3,678)	1,950 (1,025–11,032)	1.00 (1.00–1.00)	0.481	1.00 (1.00–1.00)	0.479
**Median dose of deferasirox, mg/kg (range)**	28.0 (18.9–34.1)	29.4 (17.9–34.1)	0.88 (0.75–1.04)	0.137	0.89 (0.75–1.07)	0.226
UGT1A1 *6, No. (%)				0.058		**0.028**
Wild type or heterotype	7 (77.8)	85 (95.5)	1.00 (reference)		1.00 (reference)	
Mutant homozygote	2 (22.2)	4 (4.5)	6.07 (0.94–39.16)		14.17 (1.34–150.35)	

a Creatinine elevation was defined as increased creatinine more than 50% above baseline.

b Adjusting for body weight, gender, ferritin level, and dose of deferasirox.

c Body weight was analyzed as a continuous variable. When the body weight was divided into categorical variable, creatinine elevation occurred more commonly in patients with a body weight of ≥40 kg (22.6%) compared to those with a body weight of <40 kg (3.0%) (OR = 8.48, 95% CI = 1.7–43.57, *P* = 0.010).

## Discussion

Of the total 98 patients, 15 (15.3%) developed hepatotoxicity and 9 (9.2%) had a creatinine elevation. According to the previous EPIC study enrolling 1,744 patients including pediatric cases, a relatively high cut-off value of >10×ULN was used in this study, and elevated liver enzyme levels were seen only in 0.7% of total cases [14]. In other 2 studies where a cut-off value was >5×ULN, elevated liver enzyme levels were reported in 3.8% and 7.6% of total cases, respectively [16], [30]. According to studies that have been conducted in pediatric patients, the incidence of hepatotoxicity was slightly higher. A phase II trial with 39 pediatric β-thalassemia patients reported 12.8% of elevated liver enzyme with a cut-off value of >5×ULN [19], and fluctuations of liver enzymes were reported in a recent long-term observational study with pediatric patients [31].

Our results showed that MRP2 haplotype affected the incidence of hepatotoxicity. MRP2, also known as ABCC2, is an organic anion transporter expressed at important pharmacological barriers, such as canalicular membrane of hepatocytes and epithelial cells of proximal tubules. It is involved in the biliary elimination of both endogenous and exogenous waste products [32]. When anion deferasirox is eliminated from the liver into the bile, this process seems to be partly catalyzed by MRP2 [23]. Due to this fact, it is possible that patients without wild-type allele of MRP2 haplotype are at increased risk of developing hepatotoxicity than the other patients with it.

Chronic iron overload is known to have prefibrogenic effects on the liver due to oxidative stress [33], and therefore iron overload itself can be a risk factor of hepatotoxicity in the patients receiving iron chelation therapy [34]. We did a univariate and a multivariate analysis using the ferritin level as an independent variable for predicting the risk of hepatotoxicity, but there was no association between the ferritin level and the development of hepatotoxicity. Furthermore, all patients who had hepatotoxicity showed decreased AST/ALT and bilirubin (AST/ALT ≤5×ULN and bilirubin ≤1×UNL) after discontinuation of deferasirox. These findings suggested that hepatotoxicity observed in our study was mainly associated with the administration of deferasirox.

Many previous studies have reported that creatinine elevation is one of the common adverse effects occurring at an incidence ranging from 10.0% to 39.7%, although the cut-off values were variable depending on the authors [14], [16], [31], [35].

Risk factor analyses revealed that body weight ≥40 kg and the UGT1A1*6 genotype were the risk factors of creatinine elevation. UGT1A1 encodes a UDP-glucuronosyltransferase, an enzyme of the glucuronidation pathway that transforms small lipophilic molecules, such as steroids, bilirubin, hormones, and drugs, into water-soluble, excretable metabolites [36]. Many pharmacogenetic studies have shown that UGT1A1 polymorphisms, UGT1A1*28 and UGT1A1*6 in particular, are risk factors of irinotecan-related toxicities [37], [38], [39]. Irinotecan is a prodrug that is converted to the active metabolite (SN-38), further undergoing the metabolic detoxification by hepatic UGT1A1 to an inactive metabolite [40]. Because the major metabolic pathway of deferasirox is glucuronidation by UGT1A1 [41], its toxicity could be related to the UGT1A1 polymorphism.

In our study, there was only 1 patient with UGT1A1*28/*28 homozygote, and this patient developed creatinine elevation. The distribution of UGT1A1 polymorphism is known to be variable between the ethnic groups with relatively lower UGT1A1*28 allele frequency in Asian population [37], [38], [42], [43], [44]. Our study could not demonstrate any statistical significance of UGT1A1*28 genotype, but further study is needed in Caucasian population with a somewhat higher UGT1A1*28 allele frequency.

To the best of our knowledge, this is the first study to show that side effects of deferasirox are related to the genetic polymorphisms. Hepatotoxicity and creatinine elevation were associated with MRP2 haplotype and UGT1A1*6 genotype, respectively. In conclusion, our results indicate that functional genetic variants of enzymes to metabolize and transport deferasirox are associated with drug-related toxicities. Further studies are warranted to confirm the results as the pharmacogenetic biomarkers of deferasirox.

## Supporting Information

Table S1 **Genotypes and haplotypes.**
(DOCX)
