# Supplementary material for: Pharmacogenetic Study of Deferasirox, an Iron Chelating Agent
Source: PLoS One. 2013 May 30;8(5):e64114. doi: 10.1371/journal.pone.0064114 (PMC3667856; doi:10.1371/journal.pone.0064114)
Supplement: Table S1 — Genotypes and haplotypes. (DOCX) [file pone.0064114.s001.docx]

**Supplementary Table 1. Genotypes and haplotypes.**

|  |  |  | **All** | **Hepatotoxicity** | | **Creatinine elevation** | |
| --- | --- | --- | --- | --- | --- | --- | --- |
|  |  |  | **No.** | **No. (%)** | ***p*** | **No. (%)** | ***p*** |
| **Genotype** |  |  |  |  |  |  |  |
| BCRP | 34G>A | Wild homozygote | 48 | 8 (53.3) | 1.000 | 4 (44.4) | 0.855 |
|  |  | Heterozygote | 42 | 7 (46.7) |  | 5 (55.6) |  |
|  |  | Mutant homozygote | 8 | 0 (0.0) |  | 0 (0.0) |  |
|  | 8191C>T | Wild homozygote | 95 | 14 (93.3) | 0.399 | 8 (88.9) | 0.186 |
|  |  | Heterozygote | 3 | 1 (6.7) |  | 1 (11.1) |  |
|  |  | Mutant homozygote | 0 | 0 (0.0) |  | 0 (0.0) |  |
|  | 8825C>A | Wild homozygote | 46 | 7 (46.7) | 0.893 | 5 (55.6) | 0.979 |
|  |  | Heterozygote | 42 | 8 (53.3) |  | 4 (44.4) |  |
|  |  | Mutant homozygote | 10 | 0 (0.0) |  | 0 (0.0) |  |
| MRP2 | -1774delG | Wild homozygote | 35 | 3 (20.0) | 0.336 | 3 (33.3) | 0.894 |
|  |  | Heterozygote | 51 | 9 (60.0) |  | 6 (66.7) |  |
|  |  | Mutant homozygote | 12 | 3 (20.0) |  | 0 (0.0) |  |
|  | -24C>T | Wild homozygote | 58 | 7 (46.7) | 0.874 | 5 (55.6) | 0.952 |
|  |  | Heterozygote | 38 | 6 (40.0) |  | 4 (44.4) |  |
|  |  | Mutant homozygote | 2 | 2 (13.3) |  | 0 (0.0) |  |
| UGT1A1 | -3279T>G | Wild homozygote | 52 | 9 (60.0) | 0.526 | 6 (66.7) | 0.756 |
|  |  | Heterozygote | 43 | 5 (33.3) |  | 3 (33.3) |  |
|  |  | Mutant homozygote | 3 | 1 (6.7) |  | 0 (0.0) |  |
|  | -39insTA | Wild homozygote | 77 | 12 (80.0) | 0.998 | 7 (77.8) | 0.843 |
|  |  | Heterozygote | 20 | 3 (20.0) |  | 1 (11.1) |  |
|  |  | Mutant homozygote | 1 | 0 (0.0) |  | 1 (11.1) |  |
|  | 211G>A^a^ | Wild homozygote | 65 | 9 (60.0) | 0.617 | 4 (44.4) | 0.130 |
|  |  | Heterozygote | 27 | 6 (40.0) |  | 3 (33.3) |  |
|  |  | Mutant homozygote | 6 | 0 (0.0) |  | 2 (22.2) |  |
|  | 686C>A | Wild homozygote | 95 | 14 (93,3) | 0.399 | 9 (100.0) | 0.999 |
|  |  | Heterozygote | 3 | 1 (6.7) |  | 0 (0.0) |  |
|  |  | Mutant homozygote | 0 | 0 (0.0) |  | 0 (0.0) |  |
| UGT1A3 | 17A>G | Wild homozygote | 88 | 14 (93,3) | 0.627 | 8 (88.9) | 0.925 |
|  |  | Heterozygote | 10 | 1 (6.7) |  | 1 (11.1) |  |
|  |  | Mutant homozygote | 0 | 0 (0.0) |  | 0 (0.0) |  |
|  | 31T>C | Wild homozygote | 54 | 9 (60.0) | 0.587 | 6 (66.7) | 0.825 |
|  |  | Heterozygote | 41 | 5 (33.3) |  | 3 (33.3) |  |
|  |  | Mutant homozygote | 3 | 1 (6.7) |  | 0 (0.0) |  |
|  | 81G>A | Wild homozygote | 54 | 9 (60.0) | 0.587 | 6 (66.7) | 0.825 |
|  |  | Heterozygote | 41 | 5 (33.3) |  | 3 (33.3) |  |
|  |  | Mutant homozygote | 3 | 1 (6.7) |  | 0 (0.0) |  |
|  | 133C>T | Wild homozygote | 84 | 11 (73.3) | 0.148 | 7 (77.8) | 0.481 |
|  |  | Heterozygote | 14 | 4 (26.7) |  | 2 (22.2) |  |
|  |  | Mutant homozygote | 0 | 0 (0.0) |  | 0 (0.0) |  |
|  | 140T>C | Wild homozygote | 78 | 12 (80.0) | 0.966 | 7 (77.8) | 0.887 |
|  |  | Heterozygote | 20 | 3 (20.0) |  | 2 (22.2) |  |
|  |  | Mutant homozygote | 0 | 0 (0.0) |  | 0 (0.0) |  |
|  | 477A>G | Wild homozygote | 54 | 9 (60.0) | 0.587 | 6 (66.7) | 0.825 |
|  |  | Heterozygote | 41 | 5 (33.3) |  | 3 (33.3) |  |
|  |  | Mutant homozygote | 3 | 1 (6.7) |  | 0 (0.0) |  |
| UGT1A7 | 387T>G | Wild homozygote | 39 | 5 (33.3) | 0.673 | 2 (22.2) | 0.357 |
|  |  | Heterozygote | 42 | 8 (53.3) |  | 4 (44.4) |  |
|  |  | Mutant homozygote | 17 | 2 (13.3) |  | 3 (33.3) |  |
|  | 391C>A | Wild homozygote | 39 | 5 (33.3) | 0.673 | 2 (22.2) | 0.357 |
|  |  | Heterozygote | 42 | 8 (53.3) |  | 4 (44.4) |  |
|  |  | Mutant homozygote | 17 | 2 (13.3) |  | 3 (33.3) |  |
|  | 392G>A | Wild homozygote | 39 | 5 (33.3) | 0.673 | 2 (22.2) | 0.357 |
|  |  | Heterozygote | 42 | 8 (53.3) |  | 4 (44.4) |  |
|  |  | Mutant homozygote | 17 | 2 (13.3) |  | 3 (33.3) |  |
|  | 622T>C | Wild homozygote | 59 | 7 (46.7) | 0.256 | 3 (33.3) | 0.168 |
|  |  | Heterozygote | 31 | 8 (53.3) |  | 4 (44.4) |  |
|  |  | Mutant homozygote | 8 | 0 (0.0) |  | 2 (22.2) |  |
| UGT1A9 | -118insT | Wild homozygote | 40 | 4 (26.7) | 0.311 | 3 (33.3) | 0.436 |
|  |  | Heterozygote | 41 | 9 (60.0) |  | 3 (33.3) |  |
|  |  | Mutant homozygote | 17 | 2 (13.3) |  | 3 (33.3) |  |
| **Haplotype** |  |  |  |  |  |  |  |
| UGT1A1 |  | Wild-type | 27 | 4 (26.7) | 0.835 | 1 (11.1) | 0.375 |
|  |  | Heterotype | 53 | 9 (60.0) |  | 5 (55.6) |  |
|  |  | No wild-type | 18 | 2 (13.3) |  | 3 (33.3) |  |
| UGT1A3 |  | Wild-type | 45 | 6 (40.0) | 0.706 | 4 (44.4) | 0.94 |
|  |  | Heterotype | 45 | 7 (46.7) |  | 5 (55.6) |  |
|  |  | No wild-type | 8 | 2 (13.3) |  | 0 (0.0) |  |
| UGT1A7 |  | Wild-type | 39 | 5 (33.3) | 0.673 | 2 (22.2) | 0.357 |
|  |  | Heterotype | 42 | 8 (53.3) |  | 4 (44.4) |  |
|  |  | No wild-type | 17 | 2 (13.3) |  | 3 (33.3) |  |
| BCRP |  | Wild-type | 18 | 5 (33.3) | 0.266 | 1 (11.1) | 0.205 |
|  |  | Heterotype | 37 | 4 (26.7) |  | 6 (66.7) |  |
|  |  | No wild-type | 43 | 6 (40.0) |  | 2 (22.2) |  |
| MRP2 ^b^ |  | Wild-type | 15 | 0 (0.0) | 0.099 | 1 (11.1) | 0.586 |
|  |  | Heterotype | 49 | 5 (33.3) |  | 6 (66.7) |  |
|  |  | No wild-type | 34 | 10 (66.7) |  | 2 (22.2) |  |

^a^ When analyzing wild homozygotes with heterozygotes, patients with mutant homozygotes of UGT1A1*6 had higher risk of creatinine elevation on multivariate analysis (*P*=0.028).

^b^ When analyzing wild-type with heterotype, patients without wild-type allele were at increased risk of hepatotoxicity in univariate and multivariate anlalysis. (*P*=0.008 and *P*=0.005).
